# Standardized approaches for clinical sampling and endpoint ascertainment in tuberculous meningitis studies

**DOI:** 10.12688/wellcomeopenres.15497.2

**Published:** 2020-06-03

**Authors:** Ursula K Rohlwink, Felicia C Chow, Sean Wasserman, Sofiati Dian, Rachel PJ Lai, Lidya Chaidir, Raph L Hamers, Robert J Wilkinson, David R Boulware, Fiona V Cresswell, Arjan van Laarhoven, Tuberculous Meningitis International Research Consortium

**Affiliations:** 1 Division of Neurosurgery, Department of Surgery, Neuroscience Institute, University of Cape Town, Cape Town, 7700, South Africa; 2 Weill Institute for Neurosciences and Departments of Neurology and Medicine (Infectious Diseases), University of California, San Francisco, USA; 3 Division of Infectious Diseases and HIV Medicine, Department of Medicine, University of Cape Town, Cape Town, South Africa; 4 Wellcome Centre for Infectious Diseases Research in Africa, Institute of Infectious Disease and Molecular Medicine and Department of Medicine, University of Cape Town, Observatory, Cape Town, South Africa; 5 Infectious Disease Research Center, Faculty of Medicine, Universitas Padjadjaran, Bandung, Indonesia; 6 Department of Neurology, Faculty of Medicine, Universitas Padjadjaran/Hasan Sadikin Hospital, Bandung, Indonesia; 7 The Francis Crick Institute, Midland Road, London, NW1 1AT, UK; 8 Department of Infectious Diseases, Imperial College London, London, UK; 9 Department of Biomedical Sciences, Faculty of Medicine, Universitas Padjadjaran, Bandung, Indonesia; 10 Eijkman-Oxford Clinical Research Unit, Jakarta, Indonesia; 11 Faculty of Medicine, University of Indonesia, Jakarta, Indonesia; 12 Nuffield Department of Medicine, Centre for Tropical Medicine and Global Health, University of Oxford, Oxford, UK; 13 University of Minnesota, Minneapolis, MN, USA; 14 Clinical Research Department, London School of Hygiene and Tropical Medicine, London, WC1E 7HT, UK; 15 Infectious Disease Institute, Mulago College of Health Sciences, Kampala, Uganda; 16 MRC-UVRI LSHTM Uganda Research Unit, Entebbe, Uganda; 17 Department of Internal Medicine and Radboud Center of Infectious Diseases (RCI), Radboud University Medical Center, Nijmegen, The Netherlands

**Keywords:** tuberculous meningitis, sampling, immunology, metabolomics, proteomics, microbiology, imaging, outcome, endpoints

## Abstract

Tuberculous meningitis (TBM), the most severe manifestation of tuberculosis, has poorly understood immunopathology and high mortality and morbidity despite antituberculous therapy. This calls for accelerated clinical and basic science research in this field. As TBM disproportionally affects poorer communities, studies are often performed in resource-limited environments, creating challenges for data collection and harmonisation. Comparison of TBM studies has been hampered by variation in sampling strategies, study design and choice of study endpoints.

Based on literature review and expert consensus, this paper provides firstly, practical recommendations to enable thorough diagnostic, pathophysiological and pharmacokinetic studies using clinical samples, and facilitates better data aggregation and comparisons across populations and settings. Secondly, we discuss clinically relevant study endpoints, including neuroimaging, functional outcome, and cause of death, with suggestions of how these could be applied in different designs for future TBM studies.

## Introduction

Tuberculous meningitis (TBM) is the most severe form of tuberculosis ^1^, and an important subject of observational studies and clinical trials in numerous centres internationally. However, there is a clear need to further TBM research by standardising and potentially aggregating clinical and laboratory data across multiple study sites. The International TBM Research Consortium previously published recommendations for standardised clinical data collection ^2^. However, standardised methods for sample collection and processing have not been established. Moreover, the approach to TBM outcome assessment is critical for the interpretation of clinical trials. In this paper we discuss the timing and handling of clinical samples, clinically relevant study endpoints and study design, with the aim of facilitating improved future TBM studies and data sharing.

## Sample collection and processing

### Timing

***Baseline sampling.*** Baseline research samples are ideally obtained at the time of initial routine diagnostic sampling. This can either be residual material from the diagnostic work-up, or extra sample volume, depending on the patient consent and clinical circumstances (refer to ‘Ethics’ section). Corticosteroids, routinely administered to all TBM patients ^3^, exert genomic effects within hours of administration, and non-genomic effects even faster ^4^. This is relevant to immunological studies because of changes induced in gene transcription, which can influence cytokine production and cell counts. It is, therefore, important to know the time frame between corticosteroid administration and sampling. Samples should preferably be taken before or within the first hours after administration of the first corticosteroid dose. Similarly, exposure to antituberculous drugs reduces diagnostic accuracy ^5^, and the timing of pre-study treatment relative to sampling needs to be noted. Given the potential risk of delayed treatment, patients may be started on treatment before reaching the study site. There are no strong data supporting a particular cut-off for a maximum number of days of antituberculous pre-treatment for study inclusion: a shorter duration will decrease patient heterogeneity at the cost of fewer inclusions. Studies have used a maximum of two ^6^, five ( ISRCTN 15668391), six ^7^ or seven ^8^ days of pre-treatment prior to sampling as part of their eligibility criteria.

***Follow up sampling.*** The frequency and timing of follow up samples will be influenced by sample type, e.g. blood versus cerebrospinal fluid (CSF), sampling method, e.g. lumbar puncture versus ventricular drain, and whether sampling is exclusively for research or combined with clinical activities. In centres using indwelling ventricular catheters, more frequent sampling can take place, with care to maintain sterility of the catheter valves ^9^. In other centres, given the invasive nature of CSF sampling and potential associated risks, follow up CSF samples will commonly be collected during clinically indicated procedures ^9^, or at a timepoint for pharmacokinetic or drug-adverse event monitoring ^6^. In some settings, follow up CSF sampling to reassess diagnosis is routinely performed.

Pharmacokinetic sampling from blood and/or CSF is typically performed early after treatment initiation (within 2–5 days) to analyse drug exposures in the critical initial period of illness, and to maximize sample size because of high early mortality. Subsequent sampling adds information on exposure and can be performed around day 10–14 of treatment. At that point, hepatic auto-induction by rifampicin is estimated to be at 70–80% ^10^ of its maximum, which is reached at approximately day 24 ^11^.

Immunological studies found that CSF cells start to normalise already before day two ^12^, CSF cytokines before day seven ^9^ and CSF chemistry before day 7–10 ^13^. Due to high early mortality. data from later time points may be missing. Therefore, where possible and safe, we recommend an early (day 2–3) and later (day 7–14) CSF sample for both immunological and pharmacokinetic studies. In case of clinical worsening during treatment, classified as a paradoxical reaction ^14^, repeated CSF and blood sampling could provide valuable information. For immunological studies, it may also be worthwhile repeating blood sampling after corticosteroids have been tapered ^3^ or after antituberculous treatment has been completed.

### Ethics

***Consent for sampling, storage and future use.*** Ethical considerations depend on whether samples are taken specifically for research or during clinical procedures. CSF samples are most commonly collected during diagnostic and therapeutic lumbar punctures or neurosurgery. CSF collection has the rare but serious potential of bleeding, nerve damage or infectious complications. This risk, although extremely small ^15^, together with the additional discomfort, warrants careful consideration of the balance of risk and benefit when sampling is for research only, and this requires specific consent ^6^. Discomfort from research-related procedures should be minimised; for example, heparinised extension sets, which can remain *in situ* for a full day, can be used to avoid repeated phlebotomy. Collaborative studies involving the transport of patient samples may require material transfer agreements to ensure the ethical storage and processing of samples and sharing of associated outputs. See Box 1 for further considerations.

Box 1. **Considerations regarding consent.**

**General**

- Consent should cover the purpose of the research, risks and benefits, details of samples being collected, information about genetic testing, patient confidentiality, and voluntary participation. Of note, specific consent for biobanking for future research and genetic testing must be obtained. If applicable, patients must be informed that genetic data will not be shared with them, and that intellectual property generated will not accrue to them.
- In some countries it may be required to inform patients about possible shipment of samples overseas for collaborative research and appropriate material transfer agreements may be required.
- For patients with an altered level of consciousness and compromised decision-making capacity, consent can be requested from family members. Patients should be re-consented when they regain mental capacity. Consider seeking approval from local ethics committees to waive consent for participants who do not regain the ability to consent, who demise, or whose families are not contactable, as this will enhance inclusion of more severely ill patients.

**Paediatric studies**

- Age of consent and assent may differ based on local regulations ^16^.
- Consent is required from the legal guardian of minors, to ensure children are protected from potential risks.
- Assent is required from minors as developmentally appropriate, to demonstrate respect for their participation in research ^16, 17^.

### Sampling for specific purposes

Regardless of the analyte being investigated, general principles for sample handling apply ( Box 2). Recommendations for specific samples are provided in Table 1 and discussed below.

Box 2. **Practical considerations for sample collection**

**Timing**

- Baseline samples: preferably before treatment initiation; record timing relative to starting corticosteroids and antitubercular treatment.
- Follow up samples: aim to collect during routine procedures.

**Quality**

- Process swiftly after collection.
- Consider sample volume relative to collection tube. Avoid over- or under-filling tube.
- For CSF, avoid using the initial pass of the sample, and document the appearance of the sample before and after centrifugation.
- Avoid freeze-thaw cycles. Work over dry ice if necessary.

**Compartment**

- Note if CSF is from lumbar or ventricular source.
- Ventricular CSF may be obtained from TBM patients with external ventricular drains *in situ*.
- Lumbar CSF volumes may be limited in patients with spinal arachnoiditis due to the presence of exudate in the spinal subarachnoid space.

**Biobanking**

- Biobank small aliquot volumes at -80°C. If an ultralow freezer is not available at the site of collection, consider using a -20°C freezer for a short interim.
- Label with study number, date, sample type, project information.

**Table 1.** Summary of practical considerations regarding sampling in TBM.

	Factors affecting quality	Blood	CSF
Proteins	Sensitive to freeze-thawing. Prepare aliquot plan to limit the number of cycles. Haemolysed samples may be problematic.	Collect in EDTA or heparin tubes. Centrifuge within 1 h at standardised speed (i.e. 1500–3000 × g) to obtain plasma. Store in polypropylene tubes at -80°C. Aim for ≥ 100μl aliquots.	Collect preferably in polypropylene tubes. Centrifuge within 1 h at standardised speed (i.e. 1500–3000 x g) to obtain supernatant while pelleting remaining erythrocytes and leukocytes. Store in polypropylene tubes at -80°C. Aim for ≥100μl aliquots.
Metabolites	After collection, store cool (4°C) till first centrifugation performed within 1 h. Avoid samples that appear haemolysed after centrifugation.	Collect in EDTA or heparin tubes. Centrifuge within 1 h at standardised speed (1500–3000 × g) to obtain plasma. Serum is second best. Store in polypropylene tubes at -80°C. Aim for ≥ 200μl aliquots.	Collect preferably in polypropylene tubes. Centrifuge within 1 h at standardised speed (1500–3000 × g) to obtain supernatant while pelleting remaining erythrocytes and leukocytes. Store in polypropylene tubes at -80°C. Discard samples with erythrocytes >500/μl. Aim for ≥200μl aliquots.
Pharmacokinetics	Short specimen transfer using cool box and protected from light. Maximum time to processing depends on drug, isoniazid is especially unstable.	Collect in heparin or EDTA tubes for plasma or clotted for serum assays. Centrifuge at standardised speed (1500–3000 × g for 10–15 minutes). Store in cryotubes at -80°C. Aim for ≥200μl aliquots.	Collect in cryovials directly or transfer to cryovials immediately after collection. Centrifugation not essential Store in cryotubes at -80°C. Aim for ≥200μl aliquots.
Flow cytometry	If possible, perform on fresh cells as fixation can influence expression of activation markers.	Collect in heparin or EDTA-tubes. No centrifugation until processing Store at room temperature until processing <24 h. Aim for ≥100μl blood per panel.	Collect in standard tubes Centrifuge gently (i.e. 300 × g) immediately (< 1 h) after collection Resuspend pellet in RPMI supplemented with BSA. Store at 4°C until processing <24 h. Aim for ≥500μl CSF per panel.
Transcriptomics	Early stabilisation is paramount	Collect in PAXgene tubes according to manufacturer's instructions. Aim to fill tube to maximum (2.5 mL) volume. No centrifugation. Shake tube, leave at room temperature for ≥2 h. Store at -80°C up to two years.	Collect in PAXgene tubes and process as with blood. Alternatively, collect in polypropylene tubes, centrifuge gently (800 × g for 10 minutes), add 500μl Tri-Reagent or TRIzol to pellet, mix vigorously and leave for 10 minutes at room temperature before freezing. Store blood PAXgene tubes at -80°C up to two years. No data exists on processing CSF RNA samples after more than six months.
*Ex vivo* cytokine production		Collect in heparin tubes for whole blood stimulation assays or in EDTA-tubes for subsequent PBMC isolation. No centrifugation. Store at room temperature till processing in <24 h.	N/A
Microbiological studies	Perform within 24 h after lumbar puncture.	N/A	Collect >6ml in sterile tubes. Centrifuge 10–15 minutes at 3000 × g.

TBM, tuberculous meningitis; CSF, cerebrospinal fluid; h, hour; EDTA, ethylenediaminetetraacetic acid; PBMC, peripheral blood mononuclear cells; RPMI, Roswell Park Memorial Institute medium; BSA, bovine serum albumin.

**Figure 1.**
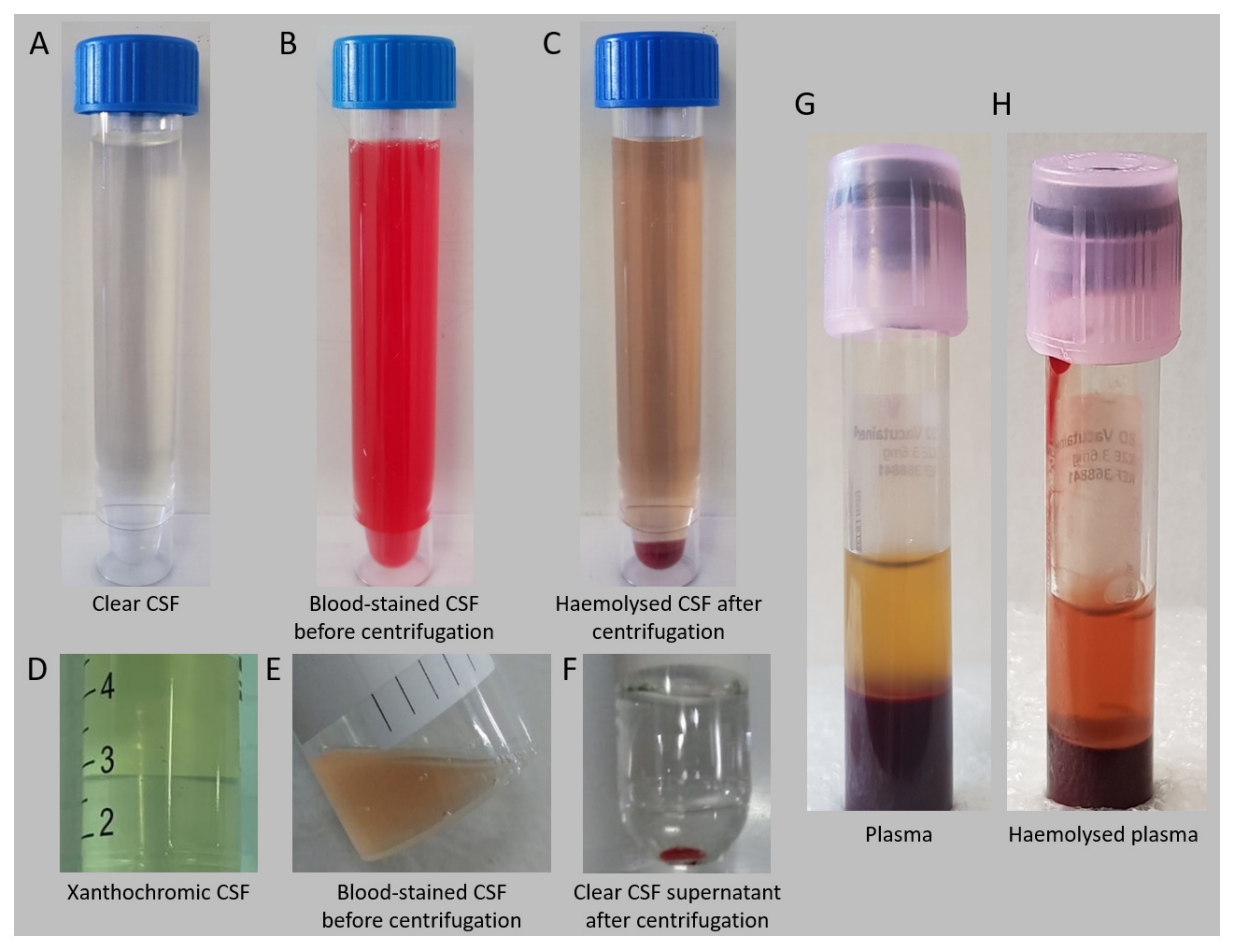
Sample characteristics. General examples of sample appearance characteristics are demonstrated. **A**: normal clear CSF; **B**: markedly blood-stained CSF (from a patient with intraventricular blood) with **C**: the same sample after centrifugation, showing partial haemolysis; **D**: xanthochromic CSF is often observed in TBM and is attributed to albumin-bound bilirubin that crossed the blood-CSF barrier; **E**: mildly blood-stained CSF with **F**: clear CSF supernatant after centrifugation; **G**: normal appearing plasma; **H**: haemolysed plasma. CSF, cerebrospinal fluid; TBM, tuberculous meningitis.

***Proteins.*** Different methods are available for proteomic analysis of blood and CSF, including tandem mass spectrometry coupled with liquid chromatography (LC-MS/MS) ^15^, antibody-based multiplex (Luminex) ^18^ or antibody-based methods using a PCR-based multiplex proximity extension assay ^19^. The concentration of CSF proteins can differ by orders of magnitude in the diseased state, which will favour choosing an analytical method with a wide dynamic range ^15^. Of note, it is necessary to validate the methods for the use of CSF, which may use a different background matrix to plasma or serum. Polypropylene rather than polyethylene tubes are preferable for storage due to their low protein binding nature. Centrifugation is important for sample purity and quality control. Samples that appear haemolysed after spinning should preferably be disregarded to avoid measuring proteins of erythrocyte origin, see Figure 1 for examples of different sample characteristics. The decline in protein concentration is less when stored at -80°C compared to -20°C ^15^. When samples are transported on dry ice, CO _2_ may enter the container’s headspace and cause acidification of the samples. After transport, it is recommended to leave the samples in a -70 or -80°C freezer to allow the CO _2_ to dissipate before samples are analysed ^20^. Freeze-thaw cycles can cause conformational changes in proteins, including cytokines, which can affect measurement by antibody-based techniques. These effects have mostly been studied in plasma and serum, and protein concentrations can both increase or decrease ^21^ because of evaporation or protein denaturation, respectively. In some instances one freeze-thaw cycle is enough to influence measured concentrations in blood ^22^, but this first cycle is generally unavoidable because most advanced measurements cannot be performed at the bedside. Data is lacking for many CSF proteins ^15^, but it is advisable to reduce the number of freeze-thaw cycles by biobanking samples in small aliquots (e.g. 100–200 μl).

***Metabolites.*** Metabolomics is a powerful technique that can be applied batchwise in small volumes of bodily fluids, which makes it suitable for studying CSF. Nuclear magnetic resonance (NMR) ^23^, gas chromatography-mass-spectrometry ^24^ and liquid chromatography-mass spectrometry ^25^ have been applied successfully on CSF of TBM patients.

Metabolomic studies gain quality with careful sampling strategies. For blood, ethylenediaminetetraacetic acid (EDTA)-plasma is preferred over serum, as EDTA inhibits enzymes and clotting is not activated ^26^. Metabolomics identified isopropanol and propylene glycol in CSF, possibly originating from disinfection prior to lumbar puncture and the CSF collection tubes, respectively. However, both are also produced in the human body and, therefore, they cannot be definitively considered artefacts ^27^. Ongoing *ex vivo*, in-tube cell metabolism affects metabolite concentrations. Blood will be especially vulnerable given the relatively high cell counts ^28^, but also some CSF metabolites can already be affected when the sample is left at room temperature for 30 minutes, and this impact increases in the next 90 minutes ^29^. We therefore recommend not delaying centrifugation by more than one hour after sampling and ideally, samples should be kept at 4°C in the meantime. Centrifugation speed can influence metabolite levels and should be consistent across all samples ^28^. Of note, concentrations of many metabolites are different (higher) intracellularly compared to plasma or CSF and haemolysis in blood samples has been shown to influence measurements and variability of blood metabolites ^26^. It is therefore preferable to obtain cell-free plasma and CSF supernatant and discard haemolytic blood and CSF samples. When CSF is sampled, preferably do not use the first tube for metabolomics as metabolite levels may be influenced by blood contamination.

When a -80°C freezer is not available in the processing lab, an acceptable strategy is to freeze samples at -20°C and transfer them to -80°C within a week to a month ^28^. In blood, the influence of a few freeze-thaw cycles seems to be relatively small ^26^. It is unclear whether sample storage at -80°C for more than 2.5 years influences metabolite concentrations ^28^.

***Pharmacokinetics.*** Pharmacokinetic sampling can follow different approaches. Intensive pharmacokinetic sampling involves collecting a large number of samples in a relatively limited number of individuals, yielding detailed information for each participant. Alternatively, sparse pharmacokinetic sampling involves a limited number of samples, which makes it feasible for use in a larger group of patients, enabling so-called population pharmacokinetic modelling. Pharmacokinetic time points depend on the approach used and the pharmacokinetic characteristics of the drugs involved. For example, catching the peak concentration with intensive pharmacokinetic sampling requires multiple samples around the time to that peak concentration; for drugs with a long elimination half-life, sampling can sometimes be limited to trough levels. It is important to note the exact timing of preceding drug doses and food intake to improve pharmacokinetic modelling, if possible including the treatment patients received before study recruitment. Where possible, take CSF and blood samples simultaneously to enable calculation of drug penetration ratios.

Stability studies specific to the research setting need to be part of the bioanalytical method validation. These studies define the sample type (EDTA or lithium-heparin plasma or serum) and the processing conditions. Samples should be processed and frozen as soon as possible after collection ^30^. Isoniazid is especially unstable with a decline in concentration seen after one hour at room temperature ^31^. Blood requires centrifugation, while CSF can be collected directly from the lumbar needle or drain into cryovials at the bedside, or aliquoted in the laboratory after collection without centrifugation. Samples can be stored at -80°C, unless stability has been validated at higher temperatures for a specific drug.

Of course, intralaboratory bioanalytical method validation needs to be performed, providing information on measures such as accuracy, precision, selectivity and limits of quantitation. Participation in an interlaboratory proficiency testing program is recommended ^32^.

Plasma assays need careful validation before they can be used to measure CSF drug concentrations ^2^. Assays for simultaneous measurement of multiple drugs make efficient use of sample volume and may need as little as 100 µL of plasma or CSF to measure concentrations of antituberculous drugs. If CSF to plasma concentration ratios are assessed based on the measured concentrations, they should be based upon drug plasma concentrations that are corrected for protein binding, as only the protein-unbound fraction of plasma is able to penetrate into the CSF ^2^.

***Flow cytometry.*** Ideally, flow cytometry is performed within hours of sample collection. However, in most field sites this is not achievable, especially not outside working hours. An alternative strategy is storing samples until flow cytometry can take place within 24 hours after sampling. Blood leukocytes thrive best when stored as whole blood in heparin or EDTA-tubes at room temperature. CSF is toxic to leukocytes, especially neutrophils and monocytes ^33^, and a delay in processing samples can cause underestimated cell counts. This can be reduced by early centrifugation, preferably within 30 minutes, and resuspension in cell-culture medium. Centrifugation must be gentle in order not to activate cells, for example at 300 × g. When the volume of CSF is known and microparticles are added to the solute, cell counts can be calculated. This strategy has been applied in TBM, storing CSF cells at 4°C until flow cytometric measurement the next day ^12^. As an alternative strategy, cells can be fixed and cryopreserved ^34^; this has been successfully piloted in CSF by one of the authors but it remains to be established whether all leukocyte populations can be successfully quantified. It is important to consider that cell fixation methods can influence later antibody-fluorochrome binding, and should be validated beforehand.

***Transcriptomics.*** The purpose of RNA-sequencing (RNA-Seq) is to elucidate differential gene expression between at least two groups, i.e. different phenotypes, or longitudinal changes following disease onset or treatment. In TBM, transcriptomics has been applied on both blood ^35^ and CSF ^36^. RNA should be stabilised immediately after collection in order to preserve RNA integrity, prevent degradation and minimise non-specific gene induction for downstream applications. For RNA stabilisation of blood, PAXgene RNA tubes (Qiagen) have been developed and used successfully ^35^. Vigorously shaking the PAXgene tube and leaving it at room temperature for at least two hours can increase RNA yield. The tube may even be left at room temperature overnight. The PAXgene tube is under vacuum for 2.5ml of blood and adding a minimum of 1ml is advised.

RNA extraction from CSF is more challenging. Leukocyte counts are approximately 20–1000 times lower than in blood and, therefore, cell-associated RNA concentrations are low. RNA released from damaged brain cells in extracellular membrane vesicles may also be measurable ^15^. RNA has been extracted successfully from CSF collected in PAXgene tubes, which contain RNA-stabilising additive, using standard extraction methods. The high protein content of lumbar CSF renders reagents containing guanidinium thiocyanate such as Tri-Reagent or TRIzol less effective in dissociating nucleic acids from their associated proteins. These reagents, however, have been applied successfully in stabilising the ventricular CSF, which has a lower protein content. The CSF needs to be spun down immediately after collection (approximately 800 × g) and TRIzol added to the pellet before cryopreservation (0.5ml). The TRIzol must be well mixed after it is added and left at room temperature for at least 10 minutes before freezing. If there is still some fluid above the pellet, TRIzol LS, which is designed for liquids, can be used. Tempus RNA tubes also contain RNA-stabilising additive and may potentially be used for lumbar CSF, but further testing of efficacy is required. Regardless of collection method, RNA-stabilised CSF may be stored in -20°C and preferably -80°C, and extraction is best performed within six months of collection for optimal RNA yield, but PAXgene tubes can be stored for up to two years. Due to the overall low RNA yield from CSF, DNase treatment during RNA extraction is highly recommended as DNA contamination will significantly impact RNA-Seq quality and specificity.

***Ex vivo induced cytokine response.*** Blood leukocyte cytokine responses are commonly studied in two different models. When peripheral blood mononuclear cells (PBMC) are isolated, calcium-binding EDTA-tubes can be used. The EDTA is washed away during the isolation of PBMC, which are subsequently resuspended in medium, resupplying the calcium ions that are necessary for the function of many immune receptors. In contrast, in a whole blood stimulation model, blood is used without centrifugation. EDTA should then be avoided and heparinised blood tubes can be used instead. Commercial heparin tubes can be contaminated with endotoxin, so it is recommended to run a nil control and regularly verify that unstimulated samples are free of cytokine production. Additionally, stimulated cells can be fixed for flow cytometry. See 12 for further details on whole blood stimulation assays.

***Microbiological studies.*** The threshold of detection is a key principle for microbiological tests. As TBM is a paucibacillary condition, the diagnostic process needs to be optimised. The volume of CSF tested is independently associated with microbiological confirmation where a volume of >6ml increases the likelihood of a positive culture and microscopy result ^37^. To improve sensitivity, centrifugation of a large volume CSF is recommended ^38^. Most laboratories concentrate CSF by centrifugation at 3000 × g for 10–15 minutes ^38^. All but approximately 2ml of supernatant should be drawn off and the pellet is resuspended by vortexing for 15–20 seconds. The resuspended cell pellet can then be divided across *M. tuberculosis* culture, acid-fast bacillus microscopy and nucleic acid amplification tests ^39^. If there is insufficient CSF available for testing, use of unprocessed CSF for Xpert MTB/RIF or repeated lumbar puncture in 48 hours is likely a better strategy. For batchwise proteomic or metabolomic studies aimed at detecting mycobacterial compounds for diagnostic purposes, both the sample pellet (cell fraction including proteins and lipids) and supernatant (metabolites) can be used.

An estimation of the *M. tuberculosis* load in the CSF may be valuable in the interpretation of immunological results, and could possibly be used as a pharmacodynamic endpoint as Xpert MTB threshold cycle predicted new neurological events (but not death) after starting treatment in one study ^40^. It should be noted that this strategy detects both live and dead bacilli. Alternatively, time-to-culture-positivity can be used.

## Clinical endpoints

### Rationale

Our current knowledge of TBM pathophysiology stems largely from observational studies. To evaluate new therapeutic and diagnostic interventions for TBM however, clinical trials are required. Definitions of relevant endpoints for smaller, exploratory trials are needed to allow progression to larger phase 3 trials, and comparison across studies. Selection of trial endpoints is influenced by study aim, expected effect size of the intervention, feasibility, budget and ability of surrogate markers to predict clinical outcomes. Here we review various clinical endpoints that have been explored in TBM studies and suggest endpoints, tools and timepoints that may improve standardisation across future trials.

### Neuroimaging

**Figure 2.**
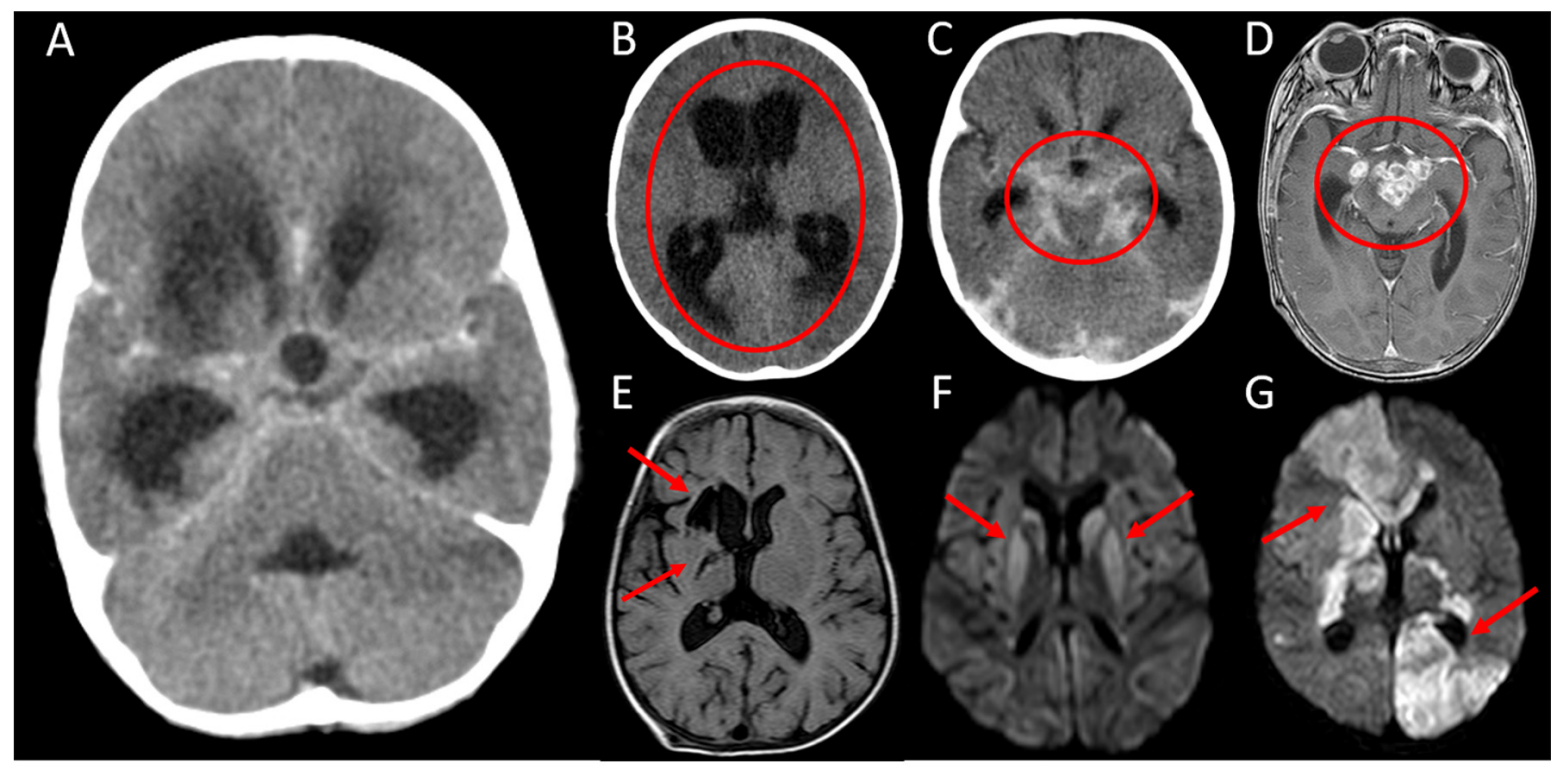
Neuroimaging findings. **A**: Axial computed tomography (CT) with contrast, demonstrating the neuroimaging triad consistent with tuberculous meningitis (TBM) - hydrocephalus, basal meningeal enhancement and a right basal ganglia infarct; **B**: Axial CT without contrast showing diffuse ventricular dilatation and transependymal fluid shift consistent with severe hydrocephalus (circled); **C**: Axial CT with contrast showing enhancing exudate within the basal cisterns and surrounding the major vessels (circled); **D**: Axial T1 - weighted magnetic resonance image (MRI) with contrast demonstrating multiple ring-enhancing lesions in the suprasellar cistern consistent with tuberculomas (circled): **E**: Axial fluid-attenuated inversion recovery (FLAIR) MRI demonstrating chronic right basal ganglia infarcts (arrows); **F**: Axial diffusion-weighted imaging (DWI) demonstrating bilateral basal ganglia high signal intensities (arrows) consistent with restricted diffusion indicating subacute bilateral infarction (the corresponding B1000 - apparent diffusion coefficient (ADC) map demonstrated low signal in the affected areas); **G**: Axial DWI demonstrating multiple areas of high signal intensity (arrows) consistent with restricted diffusion indicating acute bilateral infarction (the corresponding B1000 - ADC map demonstrated low signal in the affected areas).

The classic neuroimaging triad described in TBM is 1) basal meningeal enhancement, 2) hydrocephalus, and 3) cerebral infarction. Cerebral infarction, see Figure 2 for example neuroimaging findings, is associated with disease stage at presentation ^41, 42^ and predicts poor functional outcome and mortality ^43– 46^. The association between hydrocephalus and outcome is mixed ^47, 48^, probably because this is also a function of the treatment of raised intracranial pressure. The value of meningeal enhancement as a predictor of clinical stage or outcome is unclear ^42, 49, 50^, and enhancement may not fully resolve despite effective treatment ^51– 53^.

Pre- and post-contrast magnetic resonance imaging (MRI) is the imaging modality of choice for TBM – see Box 3. Gradient echo or susceptibility weighted imaging and MR or computed tomography (CT) angiography can be added for detection of blood and vessel pathology, if available. A contrast-enhanced fluid-attenuated inversion recovery (FLAIR) sequence may be valuable for detecting meningeal enhancement ^54^; however, the supporting data is limited, and T1-weighted imaging remains the preferred sequence ^55^. Three-dimensional MRI sequence with 1mm or thinner slices has superior yield compared to conventional two-dimensional spin echo for detecting miliary tuberculomas, including both primary lesions noted before treatment and those associated with paradoxical worsening after treatment.

Box 3. **Proposed magnetic resonance imaging (MRI) sequences**

- Pre-contrast T1 weighted imaging for normal anatomy, hydrocephalus, and for comparison with post-contrast imaging.
- T2-weighted and fluid-attenuated inversion recovery sequence (FLAIR) to detect established infarcts, hydrocephalus, and inflammation.
- Diffusion-weighted imaging (DWI) to detect acute or evolving infarcts and abscesses.
- Post-contrast T1-weighted imaging to detect breakdown of the blood brain barrier due to tuberculomas, abscesses, inflammation, meningeal and other enhancement.

If MRI is not accessible, CT with contrast can be obtained, if available. Consideration should always be given to the risk of radiation exposure with multiple CT scans, especially in paediatric patients. Additionally, CT is generally less sensitive to detect infarcts than MRI ^56^, especially those that are acute (evolving), small (e.g. lacunar infarcts), or located in the posterior fossa. If MRI has been obtained previously, subsequent imaging should also be with MRI, when feasible, as comparisons between CT and MRI are suboptimal.

***Cerebral infarcts.*** Cerebral infarcts in TBM may be subclinical and discovered only on imaging or at autopsy ^45, 57^. The onset of neurological deficits may be gradual ^45^; this, combined with the altered sensorium common in TBM, can render it challenging to detect subtle neurological deficits. Thus, for research purposes, imaging for infarcts needs to be obtained at prespecified timepoints, even in the absence of new neurological signs or symptoms. The timing depends on the clinical question. Obtaining an MRI at baseline and again at two to three months after initiation of treatment is essential, as new infarcts are frequently detected within this critical time period ^45, 53, 56^. For infarct-related questions, obtaining a third MRI toward the end of the treatment period has low yield ^53^, although late imaging may be appropriate for other questions, such as the treatment response of tuberculomas or relationship between structural abnormalities and neurocognitive impairment. Structured data extraction from neuroimaging for infarcts is important, see Table 2.

**Table 2.** Data collection for infarcts in tuberculous meningitis.

Subject	Data collected
Brain imaging modality	• Non-contrast CT • Contrast-enhanced CT • MRI without contrast • MRI with contrast
Timing of scan	• Admission/baseline • Clinical indication • Follow–up: two to three months after initiation of treatment
Type of infarct *	• Ischaemic • Haemorrhagic • Ischaemic with haemorrhagic transformation
Changes since prior brain imaging *	• New infarct identified? • Evolution of prior infarct?
Number of infarcts	• Solitary • Multifocal
In case of multifocality *	• Bilateral • In more than one vascular territory • In anterior and posterior circulation territories
Vascular territories *	• Middle cerebral artery • Anterior cerebral artery • Posterior cerebral artery • Cerebellar arteries • Vertebrobasilar perforators • Lenticulostriate perforators • Borderzone territory between two vascular territories (watershed infarcts)
Location *	• Basal ganglia • Thalamus • Internal capsule • Brainstem • Cerebellum • Subcortical white matter • Cerebral cortex
Size of infarct *	• Lacunar infarcts ** • Infarct involving >1/3 of the middle cerebral or other large artery territory • Small punctate infarcts • Combination of lacunar infarcts with larger areas of infarction
Evaluation of blood vessels	• Modality used: CT angiography, MR angiography or conventional angiography • Location of each vessel occlusion, narrowing or absence.

* More than one response allowed. ** Small subcortical infarct up to 20mm in diameter found in territories of deep penetrating arteries including basal ganglia, internal capsule, thalamus, brainstem, and corona radiata, thought to result from occlusion of a single perforating artery. CT, computed tomography; MRI, magnetic resonance imaging.

***Meningeal enhancement.*** A formal grading system for meningeal enhancement has not been developed or validated in TBM, although scoring systems have been devised for use in research studies ^58^. Poor interrater reliability of previously proposed criteria highlights the need for standardization and validation ^59^. A simple rating system that includes the presence ^60^, location (e.g. basal, sylvian fissure, convexity and ependymal) ^53^ and severity of meningeal enhancement ^61^ would allow for better standardization of radiological outcomes across studies.

***Hydrocephalus.*** Hydrocephalus is rarely seen in isolation in TBM and is typically accompanied by meningeal enhancement. Evan’s ratio—the quotient of the transverse diameter of the anterior horns of the lateral ventricles and the greatest internal diameter of the skull— can be used to standardise hydrocephalus evaluation. An Evan’s ratio of 0.3 or greater is considered abnormal ^62^, but no specific reference values exist for TBM. Similarly, a grading system for periventricular lucency (a feature of acute hydrocephalus) has been used in TBM ^61^ but not validated.

### Functional outcomes

Historically, the primary endpoint in most TBM trials has been six or nine-month mortality. This hard endpoint does not capture the full extent of TBM’s disabling neurological sequelae and more nuanced assessment of functional ability is needed.

The World Health Organization International Classification of Function (WHO-ICF) describes function in terms of impairment, activity (formerly disability) and participation (formerly handicap) ^63^. There are a variety of assessment tools for each of these domains. Properties that differentiate useful tools are validity (correlation with other tools and future outcomes), reliability (consistency of scoring between and within assessors), responsiveness to change (ability to detect meaningful change over time), and feasibility or acceptability ^64^. Context and study population must also be considered when selecting a tool.

The modified Rankin Scale (mRS) is a commonly used tool for measuring neurological disability and dependence in people having suffered a stroke or other neurological pathology. The mRS is vulnerable to interobserver variability, though this can be reduced by using a structured short questionnaire ( Table 3). Although similar to the mRS, the extended Glasgow Outcome Scale has a slightly wider range and was designed for use in brain injuries ^65^. To our knowledge it has not been used in a TBM trial and may not offer much in addition to the mRS. The Barthel Index is intended to measure performance in activities of daily living and is used to monitor progress during rehabilitation. It has been used in stroke clinical trials ^79^ and reported in a number of TBM observational studies. The only scale specifically designed for use in brain infections is the 15-item questionnaire known as the Liverpool Outcome Scale ^80^. It was designed to describe outcomes amongst children at the time of hospital discharge following encephalitis, and has predicted which children are likely to be dependent ^65^. It asks specific questions about seizures, bladder and bowel control and behaviour, which may be missed by a global scale like mRS. This has not been studied in TBM but may be a promising tool, though more time-consuming to complete. Greater detail on assessment of functional outcome and on neurocognitive impairment in adult and paediatric TBM is the topic of another article in this Tuberculous Meningitis Consortium collection ^78^.

**Table 3.** Functional outcome scales that can be applied in tuberculous meningitis (TBM).

Description and purpose	Validity, reliability & responsiveness to change *	Feasibility	Examples of use in TBM studies
**Modified Rankin Scale** Designed for stroke trials using six-point scale (0 = asymptomatic, 6 = dead). Most commonly used functional assessment.	• High validity (with other stroke scales) • Moderate reliability (stroke trials), which can be improved with use of structured questionnaire ^66^ • Limited responsiveness to change because of limited possible scores (five in survivors)	Brief yet global measure of functional recovery.	8, 67
**Barthel Index** Primarily measures independence and assists in long-term care planning in non- stroke settings. Ten-item scale delivered through a questionnaire (total score of 100) assessing ability to perform activities of daily living.	• Moderate validity • Moderate reliability • High responsiveness to change though limited by ‘ceiling’ effect ^64^	Best if based on direct observation of task but can also be done with proxy-based or telephone assessment.	68– 71
**WHO Disability Assessment Schedule** **(WHODAS) 2.0** Designed for use in adults across cultures and diseases. Questionnaire assesses six domains: cognition, mobility, self- care, interaction with others, life activities, participation. Directly linked at the level of the concepts to the International Classification of Functioning, Disability and Health (ICF).	• Moderate to satisfactory validity and reliability in European rehabilitation patients with a variety of brain disorders • Limited to moderate responsiveness to change ^72, 73^	Brief (12-item) questionnaire takes five minutes, long version (36-item) takes 20 minutes. Can be administered by telephone or proxy. Two scoring systems: simple (simple arithmetic), or a complex (domain-weighted with statistical algorithm).	Not yet used in TBM studies
**Liverpool Outcome Scale** Designed for paediatric outcomes at hospital discharge following viral encephalitis. Fifteen-item scale giving a total score of up to 75. The outcome score (range 2–5) is the lowest score for any single question.	• Moderate validity • Good inter-observer reliability ^65^ • Responsiveness to change not tested	Deemed feasible in children. Not assessed in adults yet.	To date only used in paediatric brain infection studies ^65^
**Glasgow outcome scale extended** **version for adults and paediatrics** **(GOS-E & GOS-E-peds)** Widely used in traumatic brain injury research and practice. Scale exists in adult and paediatric versions. Eight-item scale.	• Good validity in paediatric patients after severe traumatic brain injuries ^74^ • Good reliability and validity ^75^	Simple, short administration time, flexibility of administration (face-to-face, over the telephone and by post) ^76, 77^.	Not yet used in TBM studies

* ‘Validity’ describes the correlation with other assessment tools, ‘reliability’ describes the consistency of scoring between assessors (inter-assessor) and within assessors (intra-assessor), ‘responsiveness to change’ describes the ability of the tool to detect meaningful change over time ^64^. Please also refer to “Neurocognitive and functional impairment in adult and paediatric Tuberculous Meningitis” in this Tuberculous Meningitis International Research Consortium collection ^78^.

### Possible early surrogate clinical markers for longer-term outcomes

Baseline risk factors with established prognostic value for mortality include higher disease severity as indicated by British Medical Research Council (MRC) grade or low admission Glasgow Coma Scale (GCS), positive HIV-status, CSF culture positivity, and low CSF to blood glucose ratio ^8, 81^. Other markers that associated with outcome, which need validation, include normalisation of CSF leukocyte count, glucose and lactate ^82^, CSF brain injury markers ^9, 83^ or CSF metabolism ^25^. Of note, the association of baseline CSF cytokine levels with outcome show inconsistent results, as reviewed in reference ^84^.

Very limited data is available on the prognostic value of the above parameters when re-measured during the course of the disease. In the Vietnamese patient population, however, higher GCS during the course of treatment was associated with better prognosis, with good internal validity in a time-updated model ^85^.

### Ascertainment of death and most likely cause of death

Knowledge of the cause of death can improve the interpretation of pathophysiological studies. Mortality that can be directly attributed to TBM, such as brain herniation or ischemia, suggests different mechanisms compared to mortality as a consequence of neurological (i.e. bed sores caused by limb paralysis) or immunological (pneumonia caused by ‘immune paralysis’) sequelae. Post mortem studies provide the most accurate estimates of cause of death. However, feasibility can be hampered for religious, logistic or financial reasons.

Verbal autopsies, based on interviews with caregivers, can be used to estimate cause of death outside the hospital setting. The underlying assumption of verbal autopsies is that each cause of death has a set of observable features that can be accurately recognized, recalled, and reported by lay respondents ^86^. The quality of verbal autopsy information varies depending on the skills of the interviewer and memory of the respondents ^87^. Therefore, it is necessary to keep in contact with the patients or their close family members after the patient is discharged from the hospital and to obtain the information close after the patient’s death. For the purpose of establishing cause of death in patients, the WHO has released standard questionnaires. A focussed version has been used to assess the cause of death in an immunological TBM study ^12^ and is presented in Box 4.

Box 4. Verbal autopsy form for structured interview to ascertain death and most likely cause of death of a person age 15 years and above *

1.           Name of verbal autopsy interviewer:

2.           Name of verbal autopsy respondent:

3.           What is your relationship to the deceased?

             □ father □ mother □ spouse □ sibling □ other relative (specify) □ no relation

4.           What was the name of the deceased?

5.           Is the date of death known?

6.           When did the deceased die? (specify day, month and year)

7.           Where did death occur?

             □ at home □ in hospital □ at work place □ other (specify) □ don’t know

8.           Could you tell me about the illness/events that led to the death of the deceased? Did an injury or (road) accident occur?

9.           For how long was the deceased ill before passing away?

10–18.   Did the deceased have fever? Night sweats? A cough? Any breathing problems? Severe headache? A stiff, painful neck? Mental confusion or decreased level of consciousness? Convulsions? Motor impairment?

19–20.   Primary (direct) and secondary (indirect) cause of death 1 and 2 according to respondent

21.        Did the deceased finish the full TB treatment regimen?

22.        If the TB treatment was ongoing, at what stage was it? (specify no. months and days)

23.        Where did the deceased get the anti-TB drugs from?

            □ referral (study) hospital site □ private practice □ community clinic

24.        If the treatment was stopped, what was the cause?

            □   drug adverse event

            □   did not know it had to be minimum six months of treatment without interruption

            □   did not want to continue the treatment

            □   other (specify)

25.        In the last month before death, how was the daily activity of the deceased? **

            □   normal daily activity, no symptoms at all

            □   able to carry out all usual duties and activities

            □   requiring some help but able to look after own affairs without assistance

            □   requiring some help but able to walk without assistance

            □   unable to walk without assistance and unable to attend to own bodily needs without assistance

            □   bedridden, incontinent and requiring constant nursing care and attention

26.        In the final days before death, did the deceased travel to (or was hospitalized in) a hospital or health facility? ***

27.        If yes, for what reason? When? How long?

28.        What did the doctor/health provider do for the deceased?

* Adapted from the WHO ^88^ Verbal Autopsy Sample Questionnaire 3 “death of a person aged 15 years of above”, used in adapted form in a study that included cause of death in adult TBM patients in Indonesia ^12^. For patients below 15 years of age, an adoption could be made from the respective WHO template.** Adaptation from the modified Rankin Scale.*** Answer options: □ Yes (if applicable specify intensity and duration) □ No □ Don’t know.

### Endpoints for interventional trials

***Phase II trials and pharmacokinetic studies.*** Smaller early phase interventional trials are used to select optimal treatment regimens. These studies typically use surrogate markers for major clinical events as endpoints, usually measured within the first six months of study entry ( Table 4). For pharmacokinetic studies, endpoints such as drug exposure and probability of pharmacokinetic/pharmacodynamic target attainment can occur within the first week of therapy, when optimising antibiotic therapy is most critical for TBM outcomes. Non-pharmacological interventional studies are hampered by the fact that traditional biomarkers used in pulmonary TB, such as time to culture positivity, are not feasible in TBM due to low CSF bacillary load. Clinical markers of treatment response that are used include resolution of coma, the occurrence of new neurological or radiological events, and incident adverse drug reactions, which can be ascertained in the first 2–4 weeks of therapy. Statistical power for clinical efficacy endpoints can be increased by combining outcomes such as mortality and disability. These approaches may provide adequate sample sizes to evaluate dose-exposure-response relationships and define pharmacokinetic/pharmacodynamic targets for investigational antituberculosis drugs and dosing strategies ^89, 90^. Surrogate endpoint markers, such as those discussed in the section above, can potentially be used in future phase II trials, after further validation.

**Table 4.** Study design considerations for different study types.

Study type	Potential endpoints	Sample size(s) per arm	Endpoint timing (days)
**Pharmacokinetics trial**	– Probability of pharmacokinetic/pharmacodynamic target attainment. – Exposure-response relationships for efficacy and/or safety ^6, 89, 90^. – Description of pharmacokinetics in CSF and plasma ^6, 91, 92^. – Effect size of expected drug-drug interaction. – Bioequivalence of oral vs. intravenous administrations ^93^.	10–30	3–60
**Phase IIa safety trial**	– Occurrence of adverse events related to the intervention: solicited treatment-related adverse events. * – Serious adverse events. – Occurrence of immune reconstitution inflammatory syndrome and paradoxical reactions.	35–75	60
**Phase IIb efficacy trial**	– Early mortality or functional status. – Composite of early mortality and disability. – Improvement of GCS. ** – Neuroimaging results ^94^. – Disease relapse.	100–150	14–180
**Phase III efficacy trial**	– Mortality. – Functional status. – Cognitive impairment.	250–400	180–365 and beyond

* Such as occurrence of anaemia with linezolid use; hepatitis with high dose rifampicin; bleeding with aspirin. ** Other clinical parameters, and CSF parameter normalisation need to established in future studies as discussed in ‘Possible early surrogate clinical markers for longer-term outcomes’. CSF, cerebrospinal fluid; GCS, Glasgow Coma Scale

***Phase III trials.*** The requirement for long follow up periods in trials designed to evaluate functional outcomes limits feasibility; these trials take 5–10 years from conception to reporting and are extremely costly. However, conducting phase III trials for TBM are critical to inform guidelines and change practice. Because the majority of deaths in TBM occur early ^8, 67, 81^, it may be possible to design smaller efficacy trials (n = 250–350) using two-month mortality as an end-point, particularly with interventions that are expected to have a large effect size (> 30%). Two large phase III TBM trials set the primary mortality endpoint at nine months, aligning with standard of care treatment duration ^8, 67^, which increases chances of reliable follow up and will allow for better monitoring of disability in survivors. As the trajectory of neurocognitive disability in TBM is not well defined, and reducing disability is a key objective of TBM management, there is, however, also a need for longer term (1–2 years) assessments of neurocognitive function to be included in TBM trials to assess excess mortality that could still be the consequence of loss of functionality in TBM survivors.

## Conclusion

TBM causes a considerable burden of disease, necessitating a better understanding of pathophysiology and improved treatment regimens. This requires rigorous study, ideally combining comparable data from multiple centres. Based on published data and expert consensus, this paper offers practical recommendations to standardise clinical sample collection and analysis across multiple platforms. A similar evidence base for study endpoints is lacking and needs to be established, but considerations are discussed and suggestions offered.

## Ethics statement

Radiology images in Figure 2 were collected as part of routine clinical data in patients who had consented to participate in TBM research at the University of Cape Town (HREC numbers 318/2010, 200/2014 and 070/2018). The Human Research Ethics Committee approved a waiver of consent for the use of the radiology images as reference images for publication.

## Data availability

No data are associated with this article.
