# Supplementary material for: Standardized approaches for clinical sampling and endpoint ascertainment in tuberculous meningitis studies
Source: Wellcome Open Res. 2020 Jun 3;4:204. Originally published 2019 Dec 16. [Version 2] doi: 10.12688/wellcomeopenres.15497.2 (PMC7194504; doi:10.12688/wellcomeopenres.15497.2)
Supplement: Peer review history

# Peer review: Reviewer response for version 1

**DOI:** 10.21956/wellcomeopenres.16957.r38242

**Published:** 2020-04-17

**Authors:** Benedict D. Michael

**Affiliations:** 1 The Institute for Infection and Global Health, University of Liverpool, Liverpool, UK

Rohlwink and colleagues present a comprehensive summary of a proposed standardised approach to the investigation of TB meningitis which can be applied in future studies.

 They highlight the importance of testing of the cerebrospinal fluid and particularly the importance of this being obtained rapidly before corticosteroids impact critical parameters and also the utility of sampling across time-points.

 In the ethics section it may be worth including a specific mention of Material Transfer Agreements.

 Very clear guidance on the practical considerations to sampling are provided in Table 1.

 Can the authors expand on the sensitivity/specificity of the fix/cryopreserve approach relative to early traditional flow cytometry? This has many practical advantages if this were to be established.

 Can the authors please advise as to the utility of Diffusion Tensor Imaging in TBM to distinguish cytopathic from vasogenic oedema in terms of prognosticating and directing corticosteroid treatment?

 Also, is there a role for early angiography (e.g. CTA/MRA) in the prediction of those at risk of subsequent cerebral infarcts, in whom prophylactic antiplatelet might be considered? Especially given the consequences of cerebral infarction on outcome.

 Is there any role for patient-reported outcome measures in comparison to the routine ones described? As this may vary across the study settings.

 Otherwise, a very helpful manuscript.

Does the article adequately reference differing views and opinions?

Yes

Are all factual statements correct, and are statements and arguments made adequately supported by citations?

Yes

Is the Open Letter written in accessible language?

Yes

Where applicable, are recommendations and next steps explained clearly for others to follow?

Yes

Is the rationale for the Open Letter provided in sufficient detail?

Yes

Reviewer Expertise:

Neurological Infectious Disease

I confirm that I have read this submission and believe that I have an appropriate level of expertise to confirm that it is of an acceptable scientific standard.

# Response

**Published:** 2020-05-22

**Authors:** Arjan van Laarhoven

**Affiliations:** Radboud University Nijmegen Medical Centre, The Netherlands

Thank you - Dr William P. Howlett and Dr Benedict D. Michael - for your critical appraisal and for raising many interesting points. Some of the more specific issues we choose to answer below.

 We appreciate the reviewer’s insightful questions on the imaging section of the paper. 

 Data are extremely limited regarding the utility of advanced imaging (e.g., diffusion tensor imaging, DTI) in guiding the management of TBM. A few studies have examined the association of microstructural white matter injury on DTI with CSF inflammatory markers, expression of matrix metalloproteinase-9, and cognitive function (Gupta et al. J Neurol Sci 2008; Yadav et al. Acad Radiol 2010). Changes in DTI in response to anti-TB therapy have also been explored. However, to our knowledge, no studies have been published investigating the utility of DTI to distinguish cytopathic from vasogenic edema and to direct glucocorticoid therapy. 

 The handful of studies that have performed CTA or MRA in patients with TBM have observed a high prevalence of abnormal intracranial vessels. One study (n=67) found that a higher proportion of patients with abnormal MRA compared with normal MRA had evidence of a cerebral infarct on an MRI from the same timepoint (Kalita et al. Acta Radiol 2012). Among 18 patients in this study, 5 of 12 patients (42%) with abnormal MRA at baseline developed new infarcts on an MRI obtained at 3 months compared with 0 of 6 patients with a normal MRA. These results, albeit from a small sample, suggest that patients with an abnormal baseline MRA may benefit most from prophylactic antiplatelet therapy. However, in this same study, a large number of patients had an infarct without corresponding MRA abnormalities, which may speak to the limitations of angiography in identifying small vessel disease that may also be treated with antiplatelet therapy. Small trials examining the benefit of aspirin in TBM have found a trend toward a reduction in brain infarcts in patients who have received aspirin (Misra et al. J Neurol Sci 2010, Mai et al. eLIFE 2018). Whether a baseline CTA or MRA can be used to risk stratify patients and identify those who might benefit the most from antiplatelet therapy is an open question that merits further investigation.

# Peer review: Reviewer response for version 1

**DOI:** 10.21956/wellcomeopenres.16957.r38243

**Published:** 2020-04-01

**Authors:** William P. Howlett

**Affiliations:** 1 Department of Internal Medicine, Kilimanjaro Christian Medical Centre, Moshi, Tanzania

While I have read the paper as a whole, I will confine my comments to the second objective of the review “ **clinically relevant study endpoints”** starting on **Page 7 **at **“Clinical Endpoints”** and ending on **Page 13** at the end of “ **Conclusion”.**  My comments are under the following headings 

1. **General Comments**
2. **Specific comments/suggestions**
3. **Detailed review/comments/suggestions highlighted  ** 

**General Comments**

 This “editorial” Open Letter is extremely well written comprehensive and timely addressing the need for a standardised approach for methodologies for clinical sampling and clinical endpoint ascertainment’s for studies in tuberculous meningitis. This informed standardised approach is particularly important for TBM because of its late clinical presentation, difficulties in clinical laboratory and radiological diagnostic certainty coupled with its poor clinical outcome and prognosis. TBM because of its chronic and prolonged clinical course more than any other form of meningitis illustrates a need for a standardized methodological approach in future studies in order to better understand and optimise its treatment and management.  The authors emphasise the considerable burden of TBM and the need for a better understanding of its pathophysiology and improved treatment regimes. This requires rigorous study ideally combining data from multiple centres.  The present paper offers practical recommendations to standardise clinical sample collection and analysis across multiple platforms. I agree that the study endpoints for clinical, neuroradiological and functional outcomes are clearly presented and well discussed in the paper with preferences suggested and recommend that the paper should be published with minor changes listed below.

**Specific Comments**

 Minor suggestions are as follows

1. Page **9,** Paragraph heading **Hydrocephalus**. Line 3. *Evan’s ratio*.  A description of *Evan’s ratio *and its grading into mild ,moderate and severe would be useful to the reader.
2. Page **11** Paragraph heading: **Possible early surrogate markers for long term outcomes.** Line 3. The term High Medical Research Council (MRC) grade is used here incorrectly as it measures muscle power on a scale of 0-5 with 5 being normal. The correct term is British Medical Research Council stage (MRC 1-3).* with 3 being the most severe.
3. Page 11, Paragraph heading **Ascertainment of death and most likely cause of death.**  As patients < 15 yrs. are the main age group affected with TBM perhaps a short comment here on whether the very detailed verbal autopsy form/questionnaire available for the those TBM patients aged 15 yrs. and above could possibly be adapted/used for those <15 yrs.

**Detailed Review/Comments**

**Clinical endpoints. page 7, Neuroimaging pages. 7, 8 and 9.**

 The need for clear clinical end points for the standardization of intervention study outcomes is well justified. Neuroimaging is particularly well covered in this paper addressing as it does the classic triad in TBM of basal meningeal enhancement, hydrocephalus and cerebral infarction all of which are illustrated clearly in Fig 2. While the use of computerised tomography (CT) with contrast is discussed including its limitations a clear preference for magnetic resonance imaging (MRI) is indicated not least because of increased sensitivity to detect infarcts. Proposed MRI sequences are presented in Box 3. The indication for neuroimaging at baseline and repeated again 2-3 months after the initiation of treatment is emphasised the latter in order to detect subclinical neurological sequalae e.g. infarcts or hydrocephalus. The possibility of a third MRI at the end of treatment is mentioned to answer specific questions related to structural CNS damage and cognitive function and the outcome of tuberculomas Cerebral infarction is known to be associated with a poor prognosis and a very detailed list of end point data are presented in Table 2.

 Meningeal enhancement is mentioned and the need for a standardized rating system which is currently unavailable with the authors proposing a simple system based on presence, location and severity. Hydrocephalus is one of most frequent major neurological complications in TBM  and has a poor prognosis. While the authors suggest the Evan’s ratio for the standard evaluation of hydrocephalus the method of measurement is not described. A description and its grading into mild ,moderate and severe would be useful to the reader. *Evan’s ratio: maximal width of frontal horns/maximal width of inner skull >0.30.*  A potential grading system for periventricular lucency is also mentioned but not described as it is considered to less useful prognostically.

 The indications for neuroimaging as endpoints in TBM studies are very clearly addressed and stated with a preference for MRI over CT and including preferred MRI sequences and the optimum timing of scanning. The authors include a Table with a detailed list of itemized end points for infarction. They also propose a simple scale for meningeal enhancement based on presence, location and severity. Mention is made of the measurement of the degree of hydrocephalus by a recognised scale *Evan’s ratio.*

***Suggestion***  Page 9, Paragraph heading **Hydrocephalus**. Line 3 *Evan’s ratio*. A description of *Evan’s ratio *and its grading into mild ,moderate and severe would be useful to the reader.

**Functional outcomes. pages 10, 11**

 The authors make a very valid point that the standard primary end points for most TBM trials to date have been 6 or 9 month mortality. They emphasise the need for the inclusion of a method for assessment of TBM morbidity, in particular functional ability as measured in terms of disability and handicap. In order to achieve this they have listed, described and evaluated the main available functional outcome scales in Table 3. These scales are evaluated under a number of properties including validity, reliability, responsiveness to change and feasibility or acceptability, terms which  are defined in the subtext in Table 3. In their evaluation of the different scales a first mention is made of the most commonly used scale i.e. the modified Rankin Scale (mRS). Its use in measuring disability in stroke and other neurological disorders is described in particular its high validity, feasibility and reference to its previous use in TBM while also mentioning a vulnerability to interobserver variability some which is modifiable. While the extended Glasgow outcome scale has been used more in brain injuries it is considered unlikely to offer more than (mRS) and has not been used in TBM. The Barthel Index is designed to measure performance in activities of daily living and its progress over time particularly during rehabilitation but has not been used to date in TBM studies. The WHO disability assessment schedule is designed for adults and has not been used in TBM. The Liverpool Outcome Scale (LOS) is discussed in more detail as it was designed to describe outcomes amongst children at discharge from hospital following encephalitis and specifically answers questions concerning seizures, continence and behaviour, complications that occur post TBM and not addressed by the more commonly used (mRS). The LOS is described as valid, reliable, simple and designed to identify neurological disorders missed by (mRS). Limitations include that it not yet assessed In TBM and is time consuming to apply. For greater detail on assessment of functional outcome and neurocognitive impairment the reader is referred at the end of the discussion to a specific article written by the same TBM consortium which is currently under review and titled “neurocognitive and functional impairment in adult and paediatric TBM” .

 The paper addresses the importance of measurement of functional outcomes after TBM and briefly discusses and assess the main scales available to document and measure those outcomes. While it identifies the modified Rankin Scale (mRS), the most commonly used scale as valid, moderately reliable and brief to apply, it favours the Liverpool Outcome Scale as being equally valid, reliable and simple but as being more comprehensive in ascertainment of neurological sequalae of TBM whilst aware of its limitations that it not yet assed In TBM and is time consuming to apply. For a more detailed discussion the reader is referred for to a referenced article currently under review.

**No Suggestions**

**Possible Early Surrogate clinical markers for longer term outcomes. Page 11.**

 The authors list possible early surrogate clinical markers for longer term outcomes. They itemise the base line risk factors with known or proven prognostic value for increased mortality which include higher disease severity as follows:

1. High Medical Research Council (MRC) grade*
2. Low admission Glasgow Coma Scale
3. HIV Seropositivity
4. CSF TB culture positive
5. Low CSF/Blood glucose ratio

Other markers are mentioned with very limited data on their prognostic value in TBM in particular when remeasured during the course of the disease but would need validation. These include

1. Normalization of CSF: leukocyte count, glucose, lactate
2. CSF Biomarkers of brain injury and metabolism including cytokine levels.

The authors have correctly listed some of the main early surrogate clinical markers for long term outcomes in TBM. These include level of consciousness as measured by Glasgow Coma Scale (GCS 3-15) and the presence of neurological disorder and alteration in GCS as measured by the Medical Research Council scale (MRC 1-3).* The role of HIV in TBM and its deleterious effect on prognosis is listed and  the poor prognostic burden of high mycobacterial load in CSF as manifested by CSF positive culture and low CSF glucose levels. Other CSF biomarkers assessing the course of the infection are mentioned but need validation.

**Suggestion**

 Page **11** Paragraph heading: **Possible early surrogate markers for long term outcomes.** Line 3 The term High Medical Research Council (MRC) grade is used here incorrectly as it measures muscle power on a scale of 0-5 with 5 being normal. The correct term is British Medical Research Council stage (MRC 1-3).* with 3 being the most severe. See below

*British Medical Research Council (BMRC) staging* system.

**Stage I:**  Glasgow Coma Scale  of 15 with no focal neurologic signs. 

**Stage II:**  Glasgow coma scale 11-14. signs of meningeal irritation, slight or no alteration of sensorium , minor neurological deﬁcit (like cranial nerve palsies) or no deﬁcit

**Stage III:** Glasgow coma scale = or <10. severe alteration of sensorium, convulsions, focal neurological deﬁcit, involuntary movements

**Ascertainment of death and most likely cause of death.  Page 11.**

 The authors discuss the importance of knowing the cause of death and its role in improving the interpretation of pathophysiological studies. They mention clinical examples of different causes of  mortality in TBM e.g. 1) herniation or ischaemia  2) pressure sores  3) concurrent immunosuppression. While it is evident that post-mortem autopsy studies are the gold standard,  they acknowledge that for religious, logistical or financial reasons they may not occur. Verbal autopsies based on interviews with care givers can be used as an alternative to estimate the cause of death outside the hospital. This assumes that each cause of death has a set of observable features that can be accurately recognised, recalled and reported by respondents, The quality of the information depends on the skills of the interviewer and the memory of the respondent. After discharge close follow up with family members and patient carers is therefore recommended. For the purpose of establishing the cause of death in patients above the age of 15 yrs.  the WHO verbal autopsy questionnaire form is recommended and is presented in detail in Box 4.

 The authors adequately address the question of trying to ascertain the cause of death and outline the reasons why autopsies may not take place. The need for autopsies is discussed and an emphasis is placed on the value of a verbal autopsy. Qualifications and limitations of its use are well discussed and the detailed WHO verbal autopsy form for 15 yrs. and above is presented. However no mention is made of the use of a verbal autopsy form/questionnaire for the those TBM patients aged <15 yrs., the major group with TBM.

**Suggestion.** A short comment on whether the very detailed verbal autopsy form/questionnaire for the those TBM patients aged 15 yrs. and above could be adapted for those <15 yrs.

**Endpoints for Intervention trials. Page 12.**

 These are presented and discussed in detail in two paragraphs and study design considerations for different study types are presented clearly in Table 4.

 In phase II trials and pharmacokinetic studies potential endpoints, sample size and endpoint timing in days are listed under the different study types. In pharmacokinetic trials endpoints such as drug exposure and probability of pharmacokinetic /pharmacodynamic target attainment coupled with exposure-response relationship for efficacy and/or safety, drug dose response and optimal route of administration for bioavailability can measured. In Phase 11a safety trial the occurrence of adverse events is related to the intervention including serious clinical events and immune reconstitution inflammatory syndrome (IRIS) and paradoxical reactions. In phase IIb efficacy trials involving 100-150 patients for 14-180 day clinical markers of treatment response are used including early mortality or functional status, composite of early mortality and disability, improvement of GCS, Neuroimaging results and disease relapse. Phase III trials typically involve a large number of patients 250-400 and last from 180-365 days and beyond and are very costly. The need for these trials is emphasized as being critical to inform guidelines and change practice in TBM. Their importance is in underlined by their ability to determine overall mortality and resulting neurocognitive function and functional status.  

 The section is well written, clearly presented and summarized in Table 4.

**No suggestions**

Does the article adequately reference differing views and opinions?

Yes

Are all factual statements correct, and are statements and arguments made adequately supported by citations?

Yes

Is the Open Letter written in accessible language?

Yes

Where applicable, are recommendations and next steps explained clearly for others to follow?

Yes

Is the rationale for the Open Letter provided in sufficient detail?

Yes

Reviewer Expertise:

I am a general physician and neurologist who has worked in Africa for over 25 years. My area of expertise in clinical

I confirm that I have read this submission and believe that I have an appropriate level of expertise to confirm that it is of an acceptable scientific standard.
